# Supplementary figures and images for: Comprehensive genomic characterization of breast tumors with BRCA1 and BRCA2 mutations
Source: BMC Med Genomics. 2019 Jun 10;12:84. doi: 10.1186/s12920-019-0545-0 (PMC6558765; doi:10.1186/s12920-019-0545-0)

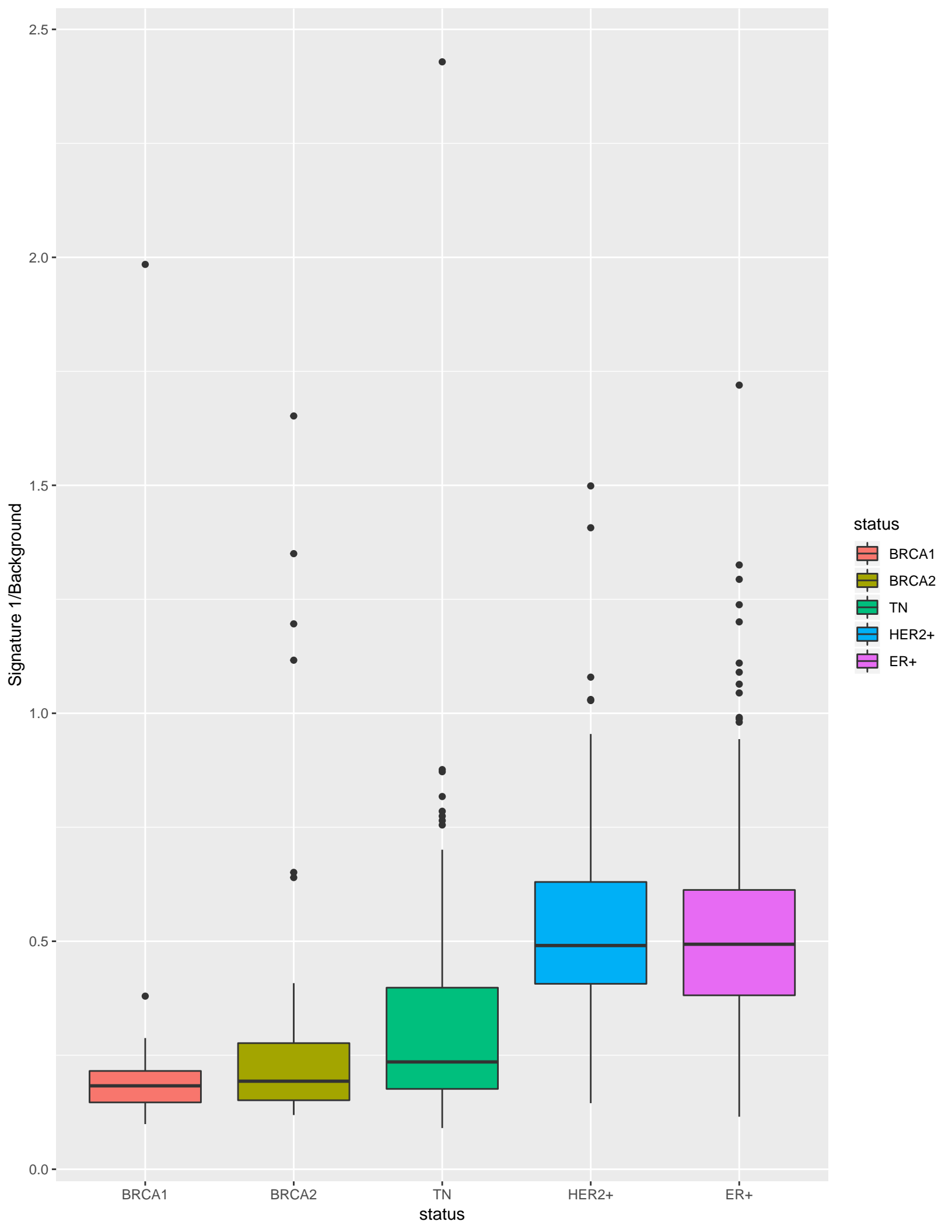

Supplement: Supplementary file 1 — Figure S1. Boxplots showing the ratio between exposures to Signature 6 (DNA CpG methylation) and the background signature, in BRCA1/2-mutated tumors and various classes if sporadic breast tumors. (PDF 6 kb) [file 12920_2019_545_MOESM1_ESM.pdf]

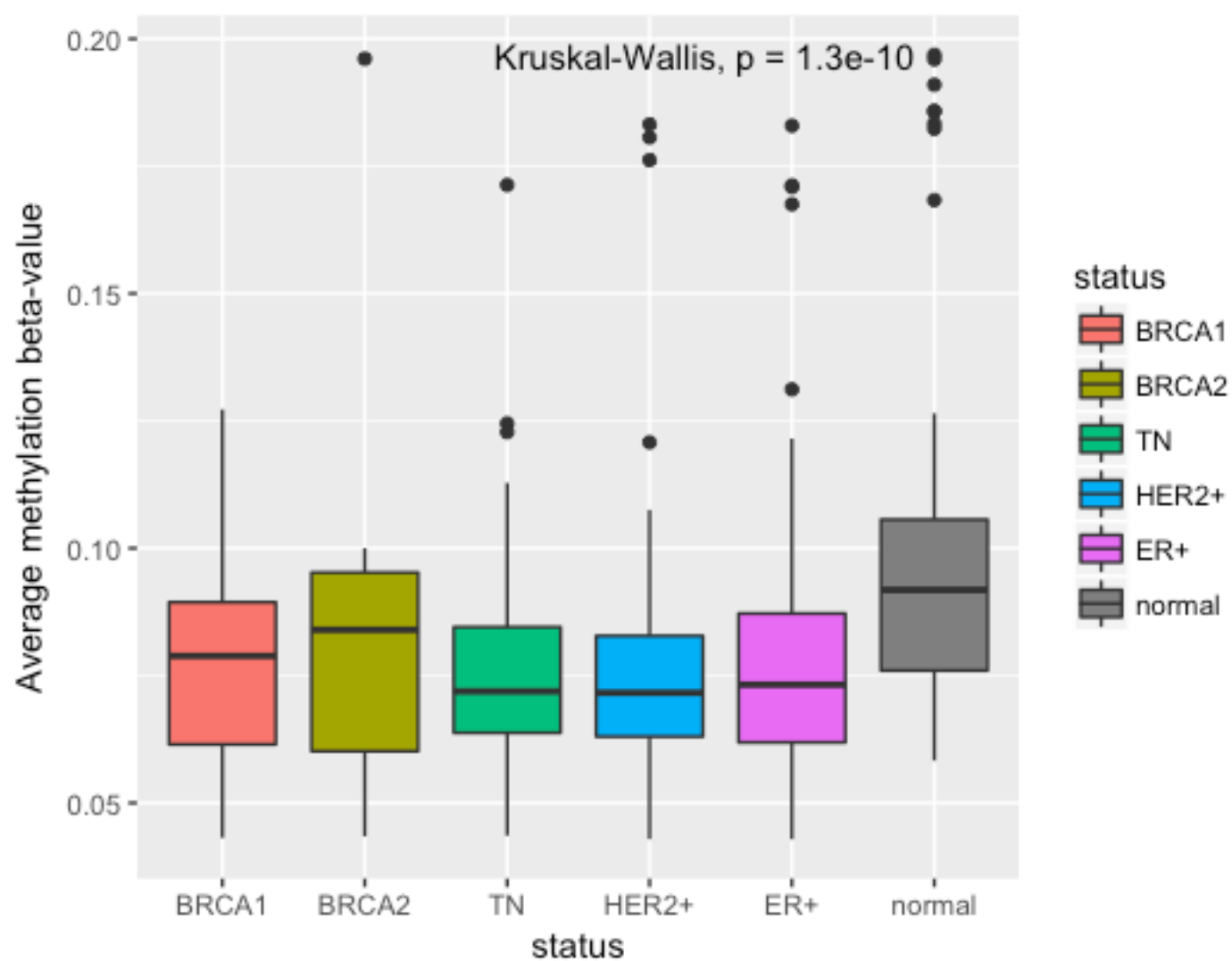

Supplement: Supplementary file 2 — Figure S2. Boxplots showing the average beta-value (extent of DNA cytosine methylation) across 3081 CpA sites, in BRCA1/2-mutated and sporadic breast tumors, based on data from TCGA. (PDF 31 kb) [file 12920_2019_545_MOESM2_ESM.pdf]

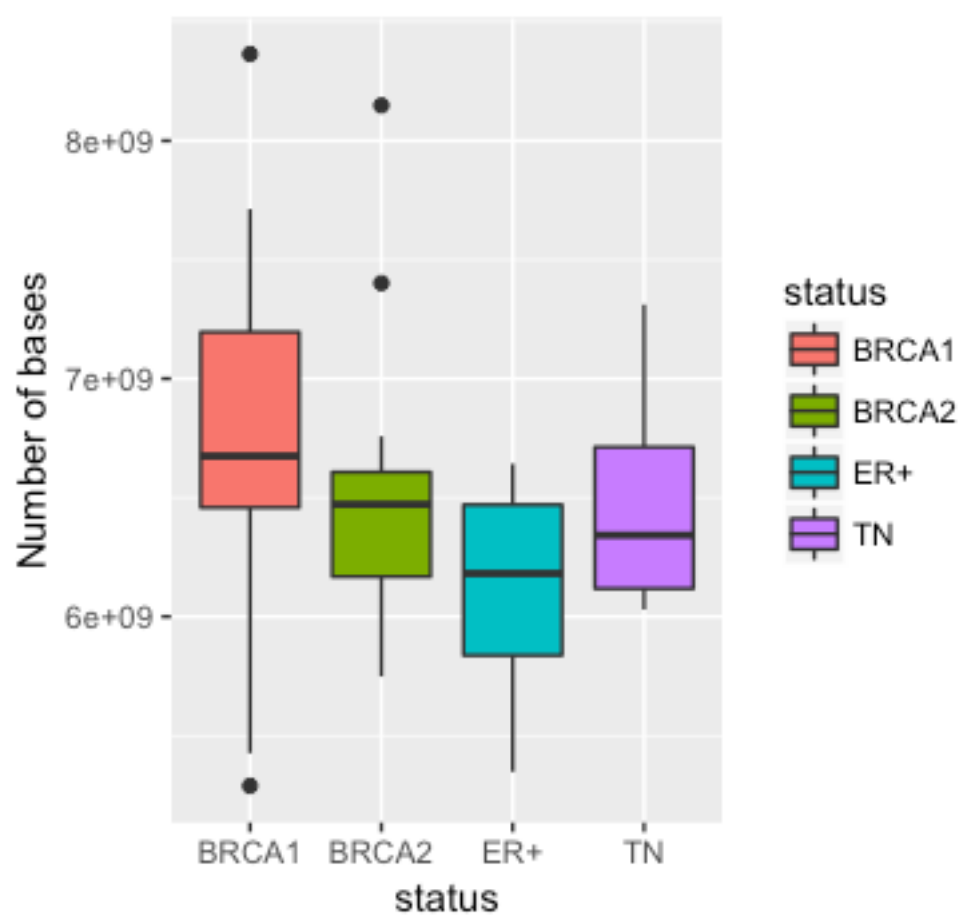

Supplement: Supplementary file 3 — Figure S3. Boxplots showing the total number of bases in the genome (accounting for copy number changes) in BRCA1-mutated, BRCA2-mutated, and sporadic breast tumors, for the dataset of 81 selected breast tumors. (PDF 19 kb) [file 12920_2019_545_MOESM3_ESM.pdf]

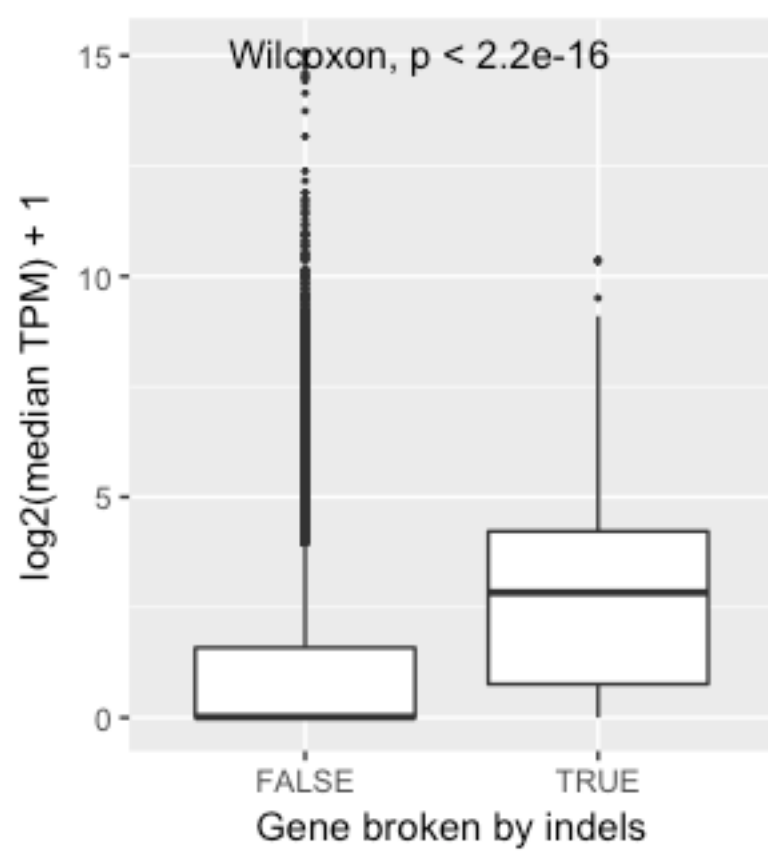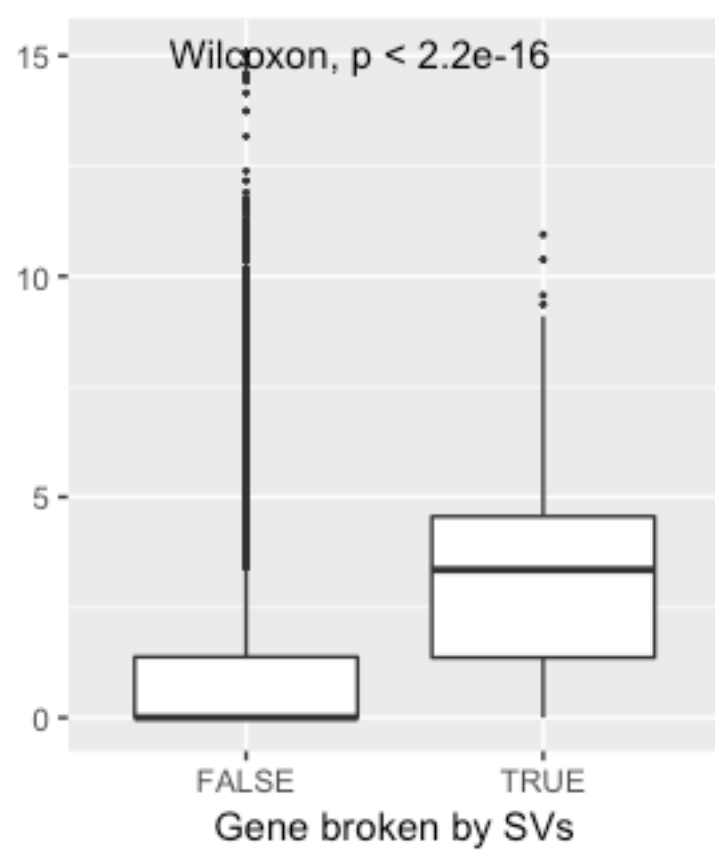

Supplement: Supplementary file 4 — Figure S4. Boxplots showing the level of expression in normal breast tissue for genes disrupted by a) indels and b) SVs in 46 BRCA1/2-mutated tumors, based on RNA-Seq data from GTEx [27]. Expression for each gene was measured as median TPM level across all breast tissue samples in the GTEx dataset. (PDF 25 kb) [file 12920_2019_545_MOESM4_ESM.pdf]

**a**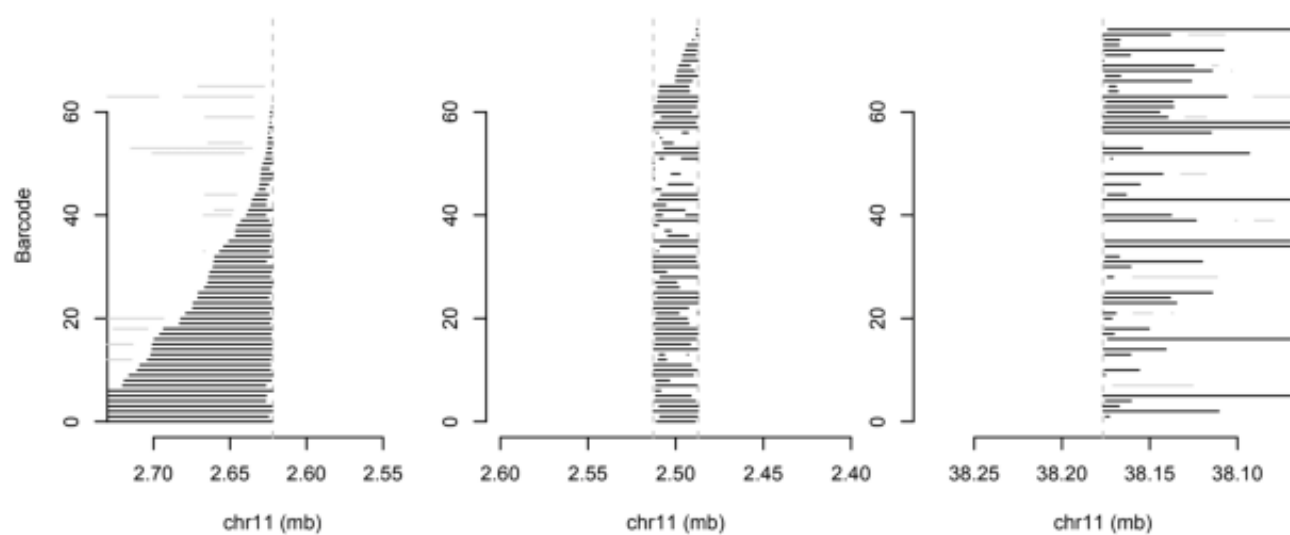**b**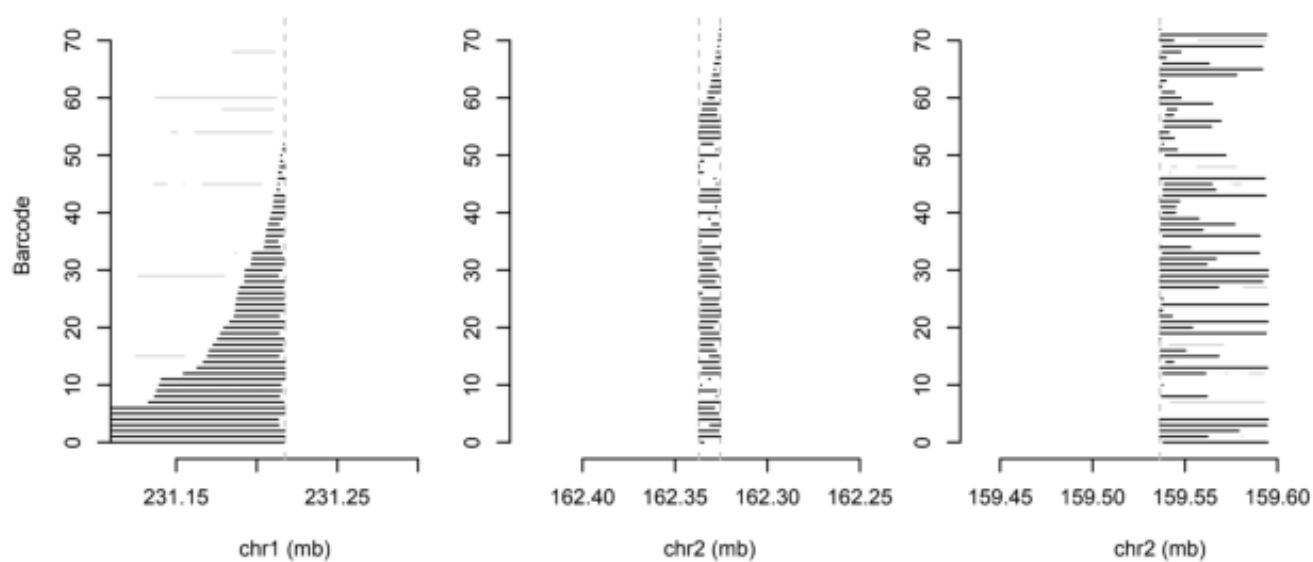

Supplement: Supplementary file 5 — Figure S5. Two complex structural variants discovered by 10X Genomics sequencing and GROC-SVs in the genome of tumor T65 containing a germline BRCA1 mutation. Inferred extent of breakpoint-supporting read clouds (corresponding to input fragments). The x-axes show chromosomal position. Each row is one read cloud (a cluster of identically barcoded linked reads). The long fragments tile across the breakpoints when ordered by their leftmost position in the left panel. (PDF 69 kb) [file 12920_2019_545_MOESM5_ESM.pdf]
